# Integrated biomarker analysis of multi-organ effects following MDMA and alcohol co-consumption in Pakistan: A pilot study

**DOI:** 10.1016/j.toxrep.2026.102324

**Published:** 2026-08-04

**Authors:** Anila Jaleel, Mahnoor Khan, Kiran Namoos, Muhammad Waleed Ibrahim, Junaid Majid Sheikh, Tahira Naseem, Shahila Jaleel, Ghulam Farid

**Affiliations:** a Department of Biochemistry, Shalamar Medical and Dental College, Lahore, Pakistan; b Biochemistry and Chemical Pathology Dept, Shaikh Zayed Postgraduate Medical Institute, Lahore, Pakistan; c Shalamar Medical and Dental College, Lahore, Pakistan; d Pathology Department, Shaikh Zayed Postgraduate Medical Institute, Lahore, Pakistan; e Shalamar Medical & Dental College, Lahore, Pakistan

**Keywords:** 3,4-Methylenedioxymethamphetamine, Ethanol, Biomarkers, Chemical and Drug Induced Liver Injury, Acute Kidney Injury

## Abstract

3,4-Methylenedioxymethamphetamine (MDMA, "Ecstasy") is a widely abused recreational drug associated with multi-organ toxicity. Objective was to compare hepatic, renal, and systemic biochemical abnormalities after acute MDMA exposure alone and with alcohol. This cross-sectional pilot study was conducted at Sheikh Zayed bin Al Nahyan and Shalamar Medical and Dental Colleges, Pakistan. The study included 24 exposed participants (ecstasy-only n = 10; ecstasy and alcohol n = 14) and 20 controls. Blood samples were analyzed for AST (<37 U/L), ALT (<63 U/L), ALP (46–116 U/L), GGT (<55 U/L), LDH (81–234 U/L), Urea (10–50 mg/dl), Creatinine (0.7–1.4 mg/dL), Uric Acid (3.5–7.2 mg/dl), and CK (39–232 U/L). Marked hepatorenal biochemical injury was predefined as ALP > 3 times the laboratory specific upper limit of normal or serum urea > 150 mg/dl. Group comparison, age and sex adjusted multivariable linear regression and ROC analyses were performed. The study group exhibited significantly elevated hepatorenal biomarkers vs. controls. Median AST, ALT and ALP were117.0, 117.5 and 439.0 U/L, equivalent to 3.16, 1.86 and 3.78 x ULN, respectively. Median urea and creatinine levels were 172.5 mg/dL and 1.42 mg/dL respectively. No significant difference was found between ecstasy-only and ecstasy and alcohol after correction. Adjusted models showed significantly higher ALP, urea, and creatinine in both study groups versus controls. RSI and STI showed high apparent discrimination (AUC 1.000 and 0.890, respectively). Acute MDMA exposure was associated with marked hepatic and renal biochemical abnormalities. Alcohol co-consumption did not independently exacerbate organ damage. The RSI and STI are novel, cost-effective, and highly accurate tools for emergency triage using routine laboratory parameters.

## Highlights

• Pilot study of multi-organ biomarker effects of MDMA–alcohol co-use.
• Hepatorenal and cardiac biomarkers were evaluated in recreational MDMA users in Lahore.
• Hepatorenal biomarkers markedly elevated in MDMA users (p < 0.005).
• No significant difference between MDMA ± alcohol; both differ markedly from controls.

## Graphical Abstract

**Figure:**
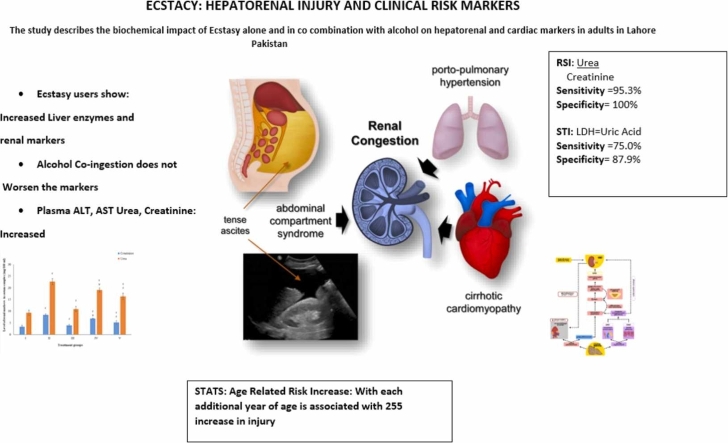

## 1 Introduction

The recreational use of drugs especially 3,4-methylenedioxymethamphetamine (MDMA, "Ecstasy") is prevalent not only in western world but also in developing countries. It is a health concern globally as well as in Pakistan. It is considered as social stigma of being elite and is frequently used by teenagers in rave parties and nightclubs. It is also now commonly used by students in high schools and colleges and is frequently distributed through illicit supply chains. The MDMA and alcohol co-consumption is reflected by epidemiological data which shows the high prevalence of their use among ecstasy users. It indicates multi-drug users, MDMA with alcohol being the most common secondary substance [11]. The prevalence of co-consumption among ecstasy users is estimated to range between 30% and 88% across various European countries [13]. MDMA is found to exert potent toxic effects on multiple organ systems, especially heart, liver and kidneys. Human clinical reports and reviews have associated MDMA exposure with acute hepatitis, marked elevations in serum alanine aminotransferase and aspartate aminotransferase, and in rare cases, fulminant hepatic failure [13], [23]. Experimental animal studies have further demonstrated that MDMA induced hyperthermia, oxidative stress and reactive metabolites may contribute to hepatocellular injury and elevated serum transaminase concentrations [14], [15]. Similarly, it causes increases in heart rate and blood pressure, leading to hypertension, tachycardia, myocardial infarction, all of which may culminate in sudden cardiac death [11], [21]. This is indicated by increased plasma levels like CK- MB, LDH and myoglobin, which are markers of myocardial stress ( [24]; Locatelli et al., 2024). These outcomes are recognized general pharmacological and toxicological risks of MDMA exposure; however, they should not be regarded as established findings in Pakistani population, as country specific clinical evidence remans limited. Renal complications of MDMA may occur through dehydration, hyperthermia, hypotension, rhabdomyolysis, hyponatremia and direct or indirect nephrotoxicity. These abnormalities may manifest as increased serum urea and creatinine concentrations, whereas CK and myoglobin may rise when clinically significant skeletal muscle breakdown occurs [17].

The alcohol consumption may modulate MDMA toxicity through the amplification of physiological stressors and pharmacokinetic interactions. MDMA undergoes o-de methylenation partly through cytochrome p450 2D6, with additional contributions from other metabolic enzymes. MDMA also acts as a mechanism-based inhibitor of CYP2D6 progressively reducing enzyme activity and contributing to greater than proportional increases in systemic exposure as the ingested dose rises [7]. Research shows that peak plasma concentration of MDMA can be increased by approximately 13% due to alcohol co consumption [13]. However, MDMA may reduce plasma levels of ethanol 9% - 15%, that suggests a complex interaction affecting initial distribution or absorption [5]. The existing literature reveals significant research gaps, particularly regarding classical cardiac injury markers and renal toxicity underscoring the importance of ongoing investigation into the long-term health consequences of this common drug abuse pattern. This manuscript explores the synergistic toxicities and MDMA alone effects, focusing on the hepatocellular, cardiovascular, and renal systems, and evaluates the clinical significance of specific biomarkers in assessing the extent of organ damage in developing country like Pakistan.

## 2 Subjects and methods

This cross-sectional study involved 24 patients as group A (Study group) and 20 healthy as group B (Control group). The study was carried out in Biochemistry departments of Sheikh Zayed bin Al Nahyan and Shalamar Medical and Dental colleges. The study was approved by the Ethical Review Board Sheikh Zayed bin Al Nahyan IRB No SZMC/TERC/Internal/443/2022. The participants of the study group were included from the parties on occasion of Eid or New year when they had bolus of ecstasy or alcohol or both. The control group were included from the non-drugs users from the staff and students of both medical schools. The participants were informed about the study details and informed consent was taken. Consent was obtained from the participants before intoxication that they are willing to provide blood samples after taking recreational drugs. Participants who appeared acutely intoxicated or lacked adequate capacity were not enrolled at that time.

Included were those between 15 and 45 years. Those who were taking ecstasy alone or in combination with alcohol were included in the study group. However, those who were not on any of these drugs and were not suffering from any acute and chronic illness were taken as controls. Excluded from study group were those with acute cardiac, renal or hepatic illness or any other endocrinological disease or with history of smoking and contraceptives use.

After taking consent, 5 ml of blood was collected by aseptic technique by the trained phlebotomist who accompanied the investigators on parties and estimation was done within 5–6 h of reported ingestion of MDMA and alcohol. The blood was centrifuged and analyzed on automatic analyzer at Sheikh Zayed bin Al Nahyan biochemistry laboratory. Serum markers for renal, cardiac and liver functions, CK, AST, ALT Alkaline Phosphatase, GGT. LDH, Urea, Creatinine and uric acid were determined on samples of study group and controls.

## 3 Statistical analysis

The data was analyzed using SPSS version 27. The qualitative variables were determined by frequency and quantitative variables were compared using Mann Whitney *U* test. Three groups were compared using Kruskal Wallis’s test. Post-hoc pairwise comparisons using Dunn’s test with Bonferroni correction done to demonstrate significance between controls and both exposure groups. Multivariable linear regression analysis was performed to adjust for confounders. Receiver operating characteristic (ROC) curve analysis was performed to evaluate the discriminatory ability of the Renal Stress Index (RSI) and Systemic Toxicity Index (STI) in differentiating patients from controls. The following exploratory composite indices were calculated from routine laboratory parameters:

Renal Stress Index (RSI)= Serum Urea/Serum Creatinine and Systemic Toxicity Index (STI) = LDH x Uric acid.

## 4 Results

The study was done on two groups i.e. study group A consisted of 24 participants and control group included 20 participants. The median age was significantly higher in group A than in control group, 28.0 years versus 24.0 years, respectively (p < 0.001). The age difference was considered a potential confounder because age may influence baseline hepatic, renal and metabolic biomarker concentrations. Therefore, younger group may have had comparatively more favorable baseline hepatic and renal biochemical values. This increases the magnitude of some unadjusted values between group differences. Age was therefore included as a covariate in multivariable linear regression analyses to adjust for its potential confounding effect.

Among participants in study group, 18 (75.0%) were male and 6 (25.0%) were female, while all participants were male (20, 100%) in the control group. Sex distribution differed significantly with females present only in group A (25% vs 0%), (Fisher’s exact test, p = 0.024). Therefore, residual confounding by sex cannot be excluded.

When Hepatorenal Biomarkers were compared in both groups, the study group exhibited significantly higher levels of all measured hepatorenal biomarkers compared to controls (p < 0.05), specifically AST (117.0 U/L), ALT (117.5.0 U/L), and ALP (439.0 U/L) and renal markers like Urea (172.5 mg/dL) and Creatinine (1.42 mg/dL). LDH, GGT, and Uric Acid were also significantly higher in study group. At the single sampling point obtained within 5–6 h of reported MDMA exposure, median CK was higher in the exposed group A than in group B, 166.0 versus 117.0 U/L but the difference was not statistically significant (p = 0.27). Table 1A

**Table 1A:** Comparison of Biochemical Markers Between Study Group A and Control Group B.

**Variables**	**Study Group A Median (IQR)** **(n = 24)**	**Control Group B** **Median (IQR)** **(n = 20)**	**p-value**
AST (U/L)	117.0 (92.0–151.0)	30.0 (27.75–40.00)	< 0.001*
ALT (U/L)	117.5 (95.25–152.0)	26.0 (20.00–35.25)	< 0.001*
ALP (U/L)	439.0 (318.75– 539.00)	78.0 (69.50–95.25)	< 0.001*
Urea (mg/dL)	172.5 (170.80–179.18)	27.0 (23.0–31.25)	< 0.001*
Creatinine (mg/dL)	1.42 (1.18– 1.64)	0.75 (0.70–0.80)	< 0.001*
LDH (U/L)	259.0 (225.75–367.50)	169.0 (151.50–192.75)	< 0.001*
GGT (U/L)	28.5 (21.75–36.00)	20.5 (17.50–24.00)	0.003*
Uric Acid (mg/dL)	7.98 (6.38–9.43)	5.99 (5.65–6.28)	0.002*
CK (U/L)	166.0 (104.75–259.75)	117.0 (106.50–193.00)	0.278

* p value < 0.05 is considered significant by Mann-Whitney *U* test

Significant differences were found across the biomarkers (p < 0.001) in ecstasy-only users (n = 10), and ecstasy and alcohol users (n = 14) compared to controls (Table 1B).

**Table 1B:** Comparison of Biochemical Markers Across Study Groups and Controls.

**Biochemical marker**	**Control Group** **(n = 20)**	**Ecstasy Only** **(n = 10)**	**Ecstasy and Alcohol** **(n = 14)**	**p-value**
AST (U/L)	30.0 (27.75–40.00)	134.0 (98.25–154.75)	99.0 (77.0–144.50)	< 0.001*
ALT (U/L)	26.0 (20.00–35.25)	107.0 (92.25–123.50)	122.5 (102.00–168.50)	< 0.001*
ALP (U/L)	78.0 (69.5–95.25)	402.0 (334.50–510.50)	450.0 (276.75–561.25)	< 0.001*
Urea (mg/dL)	27.0 (23.00–31.25)	175.15 (171.33– 182.00)	171.4 (168.48–177.80)	< 0.001*
Creatinine (mg/dL)	0.75 (0.70–0.80)	1.70 (1.46–1.89)	1.35 (1.11–1.48)	< 0.001*
LDH (U/L)	169.0 (151.5–192.75)	230.5 (201.00–249.50)	356.5 (253.00–484.50)	< 0.001*

* p value < 0.05 is considered significant by Kruskal-Wallis’s test

However, no significant difference was found for AST, ALP, and urea levels between the ecstasy and alcohol group and the ecstasy-only group. Both exposure groups significantly differed from the controls with large effect sizes (r ≥ 0.86) (Table 2).

**Table 2:** Post-hoc Dunn Bonferroni Pairwise Comparison for Significant Biomarkers.

**Biomarker**	**Group Comparison**	**Effect Size (r)**	**Adjusted p-value**
AST(U/L)	Control vs Ecstasy Only	0.84	< 0.001*
AST(U/L)	Control vs Ecstasy and Alcohol	0.74	< 0.001*
AST(U/L)	Ecstasy Only vs Ecstasy and Alcohol	0.14	1.000
ALP(U/L)	Control vs Ecstasy Only	0.79	< 0.001*
ALP(U/L)	Control vs Ecstasy and Alcohol	0.86	< 0.001*
Urea(U/L)	Control vs Ecstasy Only	0.89	< 0.001*
Urea(U/L)	Control vs Ecstasy and Alcohol	0.78	< 0.001*

* p value < 0.05 is considered significant by Dunn Pairwise comparisons with Bonferroni Test

Unadjusted analyses showed significantly higher ALP, urea and creatinine in both study groups than in controls. After adjustment for age and sex, these markers remained significantly elevated in the ecstasy group only by 534.1%, 518.5% and 101.8% respectively and in ecstasy and alcohol group by 486.6%, 492.6% and 67.3%. Age was not independently associated with these outcomes. (Table 3).

**Table 3:** Age and Sex Adjusted Multivariable Linear Regression.

**Outcome**	**Predictor**	**Adjusted β**	**95%CI**	**p value**
Log ALP	Control vs Ecstasy Only	1.847	1.514–2.183	< 0.001
	Control vs Ecstasy and Alcohol	1.766	1.439–2.092	< 0.001
	Age per year	−0.053	−0.122–0.016	0.132
	Male vs female	−0.031	−0.282–0.220	0.810
Log urea	Control vs Ecstasy Only	1.8221	1.667–1.977	< 0.001
	Control vs Ecstasy and Alcohol	1.779	1.633–1.926	< 0.001
	Age per year	0.027	−0.000–0.055	0.054
	Male vs female	−0.124	−0.241 to −0.008	0.037
Log Creatinine	Control vs Ecstasy Only	0.702	0.314–1.090	< 0.001
	Control vs Ecstasy and Alcohol	0.515	0.273–0.756	< 0.001
	Age per year	−0.006	−0.049–0.038	0.789
	Male vs female	0.043	−0.116–0.203	0.595

Separate multivariable linear-regression models were fitted for log-transformed ALP, urea, and creatinine. Controls were the reference group, and all models were adjusted for age and sex. Adjusted percentage differences were calculated as $[e^{\beta }-1]\times 100$.

The hepatic biochemical pattern was assessed using the conventional R -ratio. Using the group A median ALT of 117.5 U/L, the ALT upper limit of normal of 63 U/L, the median of ALP of 439 U/L, and ALP of upper limit of normal of 116 U/L, the group level R ratio was approximately 0.49. This suggested a predominantly cholestatic biochemical pattern.

### 4.1 R- ratio was calculated from group median values and is descriptive

Table 4A., Table 4B. showed that two composite indices were evaluated for risk stratification, which include Renal Stress Index (RSI): (Urea / Creatinine) and Systemic Toxicity Index (STI): (LDH × Uric Acid). Markedly elevated RSI and STI was demonstrated by study group participants compared to controls (p < 0.01). The RSI showed perfect apparent discrimination between study participants and control group in the derivation sample (AUC 1.000), with 100% sensitivity and specificity at a cut off of > 82.05. The STI demonstrated good apparent discrimination (AUC 0.890), with 75% sensitivity and 90% specificity at a cut off of > 1570.70 (Table 4C).

**Table 4A.** Group Level hepatic biochemical pattern using the R – ratio.

**Component**	**Calculation**	**Result**
ALT multiple of ULN	117.5/63	1.87
ALP multiple of ULN	439/116	3.78
R – ratio	1.87/3.78	0.49

Suggested biochemical pattern R < 2.

**Table 4B.** Exploratory Renal and Systemic Biochemical Indices.

**Index**	**Formula**	**Study group Median (IQR)**	**Controls Group Median (IQR)**	**p-value**
Renal Stress Index (RSI)	Urea/ Creatinine	119.10 (104.08–154.74)	35.36 (30.39–41.07)	< 0.001*
Systemic Toxicity Index (STI)	LDH × Uric Acid	2261.93 (1513.82–3213.72)	974.10 (864.15–1241.97)	< 0.001*

**Table 4C.** Apparent ROC performance of RSI and STI for distinguishing participants in study group from controls.

**Index**	**AUC**	**95% CI**	**Optimal cutoff (Youden)**	**Sensitivity**	**Specificity**
RSI	1.000	1.000–1.000*	≥ 82.05	100.0%	100%
STI	0.890	0.778–0.971*	≥ 1570.70	75%	90.0%

## 5 Discussion

The present study demonstrated that ecstasy exposure results in significant hepatocellular, cholestatic, and renal biochemical injury. Study group showed higher concentrations of ALT, ALP, urea, creatinine, LDH, GGT and uric acid. Hepatic biochemical pattern was assessed using standard R ratio [22]. An R ratio greater than 5 indicates a hepatocellular pattern, less than 2 shows cholestatic pattern and between 2 and 5 indicates mixed pattern. Using group median values R ratio was approximately 0.49, suggesting predominantly cholestatic biochemical pattern. Study by Alharbi et al., [2] shows AST and ALT serum levels were significantly increased in all abuser groups, while urea levels were significantly increased only in the combined drug abuser group, indicating specific organ damage [2]. Research studies reveal the combined hepatotoxic renal and cardio toxicity amongst drugs abusers [9], [19].

A significant finding is that the concurrent consumption of alcohol does not independently intensify ecstasy-related injury. The absence of a significant distinction between individuals using ecstasy solely and those co-consuming it with alcohol indicates that ecstasy-related toxicity is the primary contributor to organ damage in these instances. However, this finding should not be interpreted as evidence that alcohol cannot potentiate MDMA toxicity. A controlled human study reported that alcohol co-administration increased plasma MDMA concentrations by 13% (Hernández-López *et al*., 2002). The synergistic effects described in animal studies often use higher weight adjusted MDMA doses, repeated ethanol exposure and hyperthermic conditions, so species differences and the small subgroup sizes, uncertain doses and pill composition and variable exposure timing may have limited the ability to detect an interaction (Hernández-López *et al*., 2002). After adjustment for age and sex, both ecstasy only and ecstasy and alcohol groups remained significantly associated with higher ALP, urea and creatinine concentrations than controls. These findings indicate that the biochemical abnormalities were not explained by demographic imbalance, but they do not establish that alcohol produced an additional effect beyond ecstasy alone.

The standard markers for hepatocellular injury are aspartate aminotransferase (AST) and alanine aminotransferase (ALT). CK and LDH levels predict the health of cardiac functioning. The research presents innovative, cost-effective composite indices that provide enhanced diagnostic efficacy compared to individual biomarkers. Single markers like urea or LDH show only one pathway of injury, but these indices show how multiple pathways of organ injury work together. RSI (Urea/Creatinine) measures renal stress more accurately especially when dehydration or acute kidney injury (AKI) is present [1]. During volume depletion, reduced renal perfusion increases tubular urea reabsorption, producing a disproportionate increase in urea relative to creatinine. Therefore, an elevated RSI may reflect dehydration and reduced renal perfusion rather than intrinsic tubular damage [18]. Hydration status was not systematically assessed. Therefore, it could not be determined whether MDMA exposed participants were more dehydrated than controls. RSI should be interpreted as an exploratory marker of renal or pre-renal biochemical stress rather than specific marker of intrinsic injury.

CK concentrations did not differ significantly between study and control groups. This may reflect early sampling before CK reached its peak and limited muscle injury. The absence of serial CK measurements, myoglobin testing and consistently documented sampling intervals further limited interpretation. STI (LDH × Uric Acid) shows the total amount of damage to the body and stress on the metabolism. These indices, especially the RSI, is very sensitive and specific, and are useful for early clinical triage and monitoring in emergency situations. Thresholds like RSI ≥ 82.05 and STI ≥ 1570.7 could be very useful clinical signs for finding patients who are at high risk of toxicity [3].

Xu et al., [20] reports that chronic MDMA exposure may lead to valvular abnormalities in humans which are consistent with fibrotic remodeling and systemic cardiotoxic effects. The study reports **s**pecific renal dysfunction associated with MDMA (Ecstasy), which include severe hyponatremia, urinary retention, and acute kidney injury from rhabdomyolysis and malignant hyperthermia called as serotonin syndrome [4], [8].

Kern et al. (2024) reported ethanol as CNS depressant that is frequently used at gatherings. The observed elevations in ALP and transaminases are consistent with previous studies reporting ecstasy-induced hepatic necrosis [14], [13], [1]. Furthermore, the lack of a significant difference between the "Ecstasy Only" and "Ecstasy and Alcohol" groups supports the hypothesis that MDMA-related reactive metabolites are the primary drivers of organ damage, regardless of alcohol co-ingestion [12], [6]. The RSI demonstrated high apparent discriminatory performance in this pilot sample (AUC 1.000) is consistent with the clinical use of urea-creatinine ratios to identify acute renal stress in hyperthermic and dehydrated states often associated with MDMA use [13], [1]. After adjustment for age and sex, ALP, urea and creatinine remained significantly elevated in both study groups, supporting MDMA associated hepatorenal biochemical dysfunction. This agrees with recent evidence of transient increases in urea and creatinine following MDMA exposure and reported MDMA associated hepatic injury [10], [16]. However, the findings did not demonstrate an additional effect of alcohol.

The study reveals insights into the hepatorenal toxicity as a result of ecstasy and ethanol use at mass gatherings and parties in Pakistan. This study may be used as baseline to create awareness amongst masses to control drug abuse.

## 6 Conclusion

The finding of this study emphasizes that acute ecstasy exposure results in marked, multi-organ biochemical injury, characterized by a profound renal stress and cholestatic liver pattern. However, in this study the addition of alcohol did not independently exacerbate this damage, which suggests that ecstasy-related toxicity is the primary driver of injury. Th Renal Stress Index (RSI) and Systemic Toxicity Index (STI), both of which showed exceptional accuracy in identifying affected patients may be used for better management of these patients.

## 7 Limitations of the study

Despite the important findings, certain limitations were needed to be addressed to ensure the validity of the results:

1. The study comprised of a cohort of 24 subjects in study group and 20 controls. In spite of limited sample size, the high effect sizes and highly significant p-values (p < 0.001) for primary biomarkers indicate the study was sufficiently powered to capture the massive biochemical shifts caused by ecstasy.
2. There was a significant age gap between subjects in study group and controls, which was addressed by using multivariate logistic regression. It confirmed that while age is a risk factor, the organ injury observed was independently associated with ecstasy exposure and not merely a result of age variance.
3. The significant sex imbalance between groups may have introduced residual confounding and limited the generalizability of the findings.

## 8 Strengths of the study

This research possesses several distinct advantages that contribute to the field of clinical toxicology:

1. The study confirms THE RSI and STI as integrative tools that move beyond single-biomarker analysis to capture complex multi-organ interactions.
2. The RSI demonstrated a sensitivity of 100% and a specificity of 100%. Such high specificity is rare in clinical biomarkers and offers a reliable threshold for ruling in toxicity.
3. All biomarkers in this study are derived from routine, low-cost laboratory parameters making them immediately accessible in standard emergency settings, unlike expensive specialized testing,

## 9 Implications

1. Clinical Triage: The RSI cutoff > 82.05 and STI cutoff of > 1570.7 offer pragmatic "triage thresholds" to help emergency departments quickly identify high-risk patients.
2. Public Health: The finding that ecstasy alone is sufficient to cause severe organ damage independently of alcohol challenges the misconception that toxicity only occurs during "poly-substance" use, highlighting the intrinsic danger of MDMA.
3. Research Foundation: These indices represent a validated framework that can now be tested in larger external cohorts to establish global standards for monitoring drug-induced toxicity.

## Authors contribution

1. AJ MK: The conception and design of the study
2. MWI, MJ: Acquisition of data,
3. KN: Analysis and interpretation of data.
4. GF, SJ: Drafting the article or revising it critically for important intellectual content.
5. AJ, TN: Final approval of the version to be submitted.

## CRediT authorship contribution statement

**Mahnoor Khan:** Writing – original draft, Data curation, Conceptualization. **Anila Jaleel:** Writing – review & editing, Supervision, Data curation. **Shahila Jaleel:** Software, Resources, Methodology, Formal analysis, Data curation. **Tahira Naseem:** Writing – review & editing, Supervision, Data curation, Conceptualization. **Ghulam Farid:** Software, Resources, Investigation, Data curation. **Junaid Majid Sheikh:** Writing – original draft, Resources, Investigation, Data curation. **Muhammad Waleed Ibrahim:** Resources, Investigation, Data curation, Conceptualization. **Kiran Namoos:** Software, Methodology, Formal analysis.

## Financial disclosure

This research did not receive any specific grant from funding agencies in the public, commercial, or not-for-profit sectors*.*

## Declaration of Competing Interest

The authors declare that they have no known competing financial interests or personal relationships that could have appeared to influence the work reported in this paper.

## Data Availability

Data will be made available on request.
